# Constitutive modelling of hydrolytic degradation and viscoplasticity in glassy polymers: An effective temperature approach

**DOI:** 10.1016/j.ijplas.2026.104690

**Published:** 2026-06

**Authors:** Zhouzhou Pan, Huanming Chen, Laurence Brassart

**Affiliations:** Department of Engineering Science, University of Oxford, Oxford OX1 3PJ, United Kingdom

**Keywords:** Hydrolysis, Diffusion, Chemo-Mechanics, Biodegradable Polymers, PLA

## Abstract

Hydrolysis is the primary degradation mechanism in biodegradable polymers in aqueous environments, involving water diffusion and polymer chain scission. These two processes dynamically alter the composition of the polymer, significantly influencing its thermomechanical properties and deformation behaviour. In this work, we develop a constitutive modelling approach that couples water diffusion, hydrolytic chain scission and viscoplastic deformation in glassy polymers. The effect of water concentration and hydrolytic degradation on the mechanical properties is captured through an effective temperature, reflecting the reduction in glass transition temperature brought about by water uptake and the reduction in average molecular weight. The model is calibrated using experimental data for polylactic acid (PLA), including thermo-mechanical characterisation in the wet degraded state and dry undegraded state at different temperatures. Our model accurately captures the evolution of molecular weight and water concentration distributions measured experimentally, and successfully predicts the deformation behaviour at different degradation stages. The potential of the model for weakly coupled simulations is also illustrated in representative case studies. Overall, this study supports the use of the effective temperature as a practical yet physically-motivated method for capturing the effect of degradation on mechanical properties, while providing a robust tool for the design and analysis of degradable polymer devices.

## Highlights

• Thermo-mechanical characterisation of amorphous PLA during hydrolytic degradation.
• Constitutive model coupling water diffusion, hydrolysis, and viscoplasticity.
• Effect of degradation on mechanical properties well captured via an effective temperature.
• Numerical implementation in Abaqus via UMATHT and UMAT.
• Both water uptake and hydrolysis significantly impact the mechanical response.

## 1 Introduction

Biodegradable polymers, such as poly(lactic acid) (PLA), polycaprolactone (PCL) and polyhydroxyalkanoates (PHA), are a class of polymers designed to break down and eventually disappear after having fulfilled their intended function. These polymers are increasingly used in a broad range of applications including packaging, agriculture and textiles, due to growing environmental sustainability concerns (Kalita and Hakkarainen, 2023, Samir et al., 2022, Verma et al., 2024). The combination of biodegradability and biocompatibility also makes these polymers attractive for biomedical applications such as drug delivery systems, tissue engineering scaffolds, surgical sutures, and orthopaedic implants, where they eliminate the need for secondary retrieval surgery (Li et al., 2020, Kirillova et al., 2021, Hussain et al., 2024). One of the main challenges in biodegradable polymer design for load-bearing applications is to achieve simultaneous control on the degradation rate and the evolving mechanical properties. Conventional experimental trial-and-error approaches are particularly costly, considering their degradation time scales of the order of months or even years. Therefore, constitutive models that can simulate the coupled degradation and mechanical behaviour of biodegradable polymers over their service life are needed.

This work focuses on the degradation and mechanical response of PLA, which is one of the most widely used biodegradable polymers. PLA is a thermoplastic polymer with glass transition temperature $T_{g}$ in the range 55 to 65 °C, and which can be found in purely amorphous or semi-crystalline form (Farah et al., 2016). In an aqueous environment, PLA primarily degrades by hydrolysis, involving the cleavage of polymer chains by the reaction of water with ester bonds along the polymer backbone. The hydrolysis reaction can be expressed as (Laycock et al., 2017):

(1) $$\text{R–COO–R’}+{\text{H}}_{2}\text{O}\to \text{R–COOH}+\text{R’–OH}$$

where R–COO–R’ represents an ester on the polymer backbone of a PLA chain reacting with water, R and R’ represent hydrocarbon groups, and R–COOH and R’–OH are two shorter chains produced by chain scission, respectively containing carboxylic acid and hydroxyl end groups. The fundamental hydrolysed unit is the lactic acid molecule, with formula CH${}_{3}$ —CH(OH)—COOH. In general, the hydrolysis rate depends on the concentrations of ester bonds, water, and carboxylic acid end groups which act as catalyst. Other known factors impacting the degradation rate include the temperature, pH, tacticity, and crystallinity fraction (Lyu et al., 2007, Codari et al., 2012, Rodriguez et al., 2016, Woo and Wee, 2024). As degradation proceeds, the average molecular weight of the chains decreases. Eventually, short chains become mobile and diffuse into the environment, leading to mass loss. In general, diffusion of water into the polymer is faster than degradation, so that PLA predominantly undergoes a bulk erosion process, characterised by an initial reduction in molecular weight without any detectable mass loss. However, surface erosion can also occur concurrently to bulk erosion, depending on pH, temperature and specimen geometry, which can impact the ratio of diffusion and reaction timescales (Siparsky et al., 1997, Von Burkersroda et al., 2002, Laycock et al., 2017, Woo and Wee, 2024).

Exposure of PLA to an aqueous environment usually results in a decrease in the elastic modulus and yield strength (Laycock et al., 2017, Chen et al., 2023, Chen et al., 2026). This behaviour is primarily attributed to hydrolysis-induced reduction in chain length and water absorption, which both increase the mobility of the polymer chain segments (Wu and Buckley, 2004, Xu et al., 2025, Vyavahare et al., 2014). However, this molecular picture can be complicated by degradation-induced recrystallisation, which can lead to non-monotonic evolution of the elastic modulus during degradation (Saha and Tsuji, 2006, Polak-Kraśna et al., 2021). Hydrolytic degradation also leads to a ductile-to-brittle transition as the chain length falls below a critical molecular weight (Chen et al., 2026). Conversely, mechanical loads can impact the degradation rate. Literature results generally suggest that mechanical loads applied simultaneously to exposure to water accelerate the degradation rate (Fan et al., 2008, Li et al., 2010, Guo et al., 2016, Dreher et al., 2016, Li et al., 2017). In particular, we recently showed that compressive loads accelerate the degradation of amorphous PLA when the molecular weight falls below a certain threshold (Chen et al., 2026).

Modelling the degradation behaviour of biodegradable polymers at the macroscopic scale typically involves the formulation of reaction–diffusion equations tracking compositional changes. For example, phenomenological models have been proposed by Pan and co-workers to describe the concurrent evolution of the concentration of ester bonds and reaction products (short chains and monomers), accounting for mass loss, autocatalysis, distinct reaction rate constants for chain-end and internal bonds, and recrystallisation (Wang et al., 2008, Han and Pan, 2009, Gleadall et al., 2014), see also Pan (2014). Reaction–diffusion models have also described the role of water diffusion to study the interplay between surface and bulk erosion (Shockley and Muliana, 2020, Linde et al., 2022). However, these models rely on empirical expressions for the evolving concentrations of selected species and cannot predict the evolution of the molecular weight distribution. This shortcoming can be addressed using discrete chain scission models that keep track of populations of chains of different lengths and can naturally describe non-random reaction kinetics (Simha, 1941, Antheunis et al., 2009, Codari et al., 2012, Soares and Zunino, 2010). One challenge associated with discrete chain scission models is the need to solve a large system of differential equations, making them too costly for the simulation of heterogeneous degradation at macroscopic scale. Recently, we developed a two-scale reaction–diffusion framework which preserves the detailed description of discrete chain-scission models while enabling large-scale simulations of heterogeneous degradation by decoupling the description of chain scission mechanism and kinetics (Pan and Brassart, 2023). One common limitation of the aforementioned models is that they neglect the coupling of degradation with mechanics.

There have been limited attempts so far at coupling reaction–diffusion degradation models to mechanics. Most approaches borrowed concepts from continuum damage mechanics, where the elastic modulus (or elastic free energy) is taken as a decreasing function of an internal damage variable, typically related to the average molecular weight. For example, Soares et al. (2008) adopted a neo-Hookean model with degradation-dependent shear modulus. The evolution of damage was described based a model for strain-assisted degradation obtained from a maximum dissipation principle (Rajagopal et al., 2007). Muliana and Rajagopal (2012) developed a quasi-linear viscoelastic model for biodegradable polymers coupled to water diffusion. Degradation was described by taking the relaxation modulus and relaxation time as functions of the degradation state. The evolution of degradation was described using a phenomenological model assuming that both strain and water concentration impact the degradation rate, but hydrolysis was not explicitly described. Vieira et al. (2014) combined a simple hydrolysis model and the Bergström–Boyce viscoplastic model (Bergström and Boyce, 1998), neglecting water diffusion. de Castro and Fancello (2017) proposed a constitutive model for large elasto-viscoplastic deformations coupled to hydrolytic damage within an incremental variational framework. The model was able to simulate coupled effects of ductile and hydrolytic damage in complex geometries. However, the model did not include an explicit description of hydrolysis reaction and did not consider diffusion of water and degradation products. It is also worth noting that constitutive models coupling mass transport, chemical degradation and deformations have recently been developed in the context of crosslinked polymers and hydrogels (Zhou and Jin, 2020, Konica and Sain, 2020, Bahrololoumi et al., 2021, Pan and Brassart, 2022).

The objective of this work is to develop a constitutive modelling framework for coupled hydrolytic degradation, water transport, and elasto-viscoplastic deformation in amorphous glassy polymers such as PLA. To describe heterogeneous degradation, we extend our previously-proposed reaction–diffusion framework (Pan and Brassart, 2023) by including a description of water diffusion. The average molecular weight and water concentration predicted by the reaction–diffusion model are then used to estimate the mechanical properties of the wet degraded polymers. Here, we adopt the effective temperature concept initially proposed by Söntjens et al. (2012), which postulates that the behaviour of the degraded polymer at a certain temperature is the same as the behaviour of the undegraded polymer at some higher effective temperature, which is correlated to the reduction in $T_{g}$ brought about by hydrolytic degradation and water uptake. Analogous temperature-water content equivalence principles were proposed to capture the effect of water uptake on mechanical properties (Miri et al., 2009, Fabre et al., 2018, Parodi et al., 2018, Chen et al., 2023). A key advantage of our proposed approach is that the mechanical behaviour of the wet degraded polymer is captured through a single parameter. However, the model also requires a description of the temperature-dependence of the intact polymer, as well as a description of the role of water content and degradation state on $T_{g}$. The constitutive model is implemented in the finite element software Abaqus through user-defined subroutines, and its potential for guiding the design of biodegradable materials and devices are illustrated in several case studies.

In addition, a systematic experimental investigation was conducted on a commercial grade of purely amorphous PLA to inform the model development and calibrate model parameters. Degradation tests at 45 °C were conducted for up to 45 days to characterise the evolution of average molecular weight and water uptake with time. The mechanical behaviour of the polymer was also characterised, both before and after degradation. We further characterised the evolution of the glass transition temperature as a function of the water content and average molecular weight. Comparisons between the model predictions and experimental data suggest that the proposed model well describes compositional changes and deformation behaviour.

The paper is organised as follows. We present the experimental procedures and main experimental observations in Section 2. Based on the experimental results, the general consideration and assumptions of the model are outlined. In Section 3, we develop a continuum thermodynamic framework for concurrent deformation, water transport and hydrolytic degradation. Specific constitutive equations are presented in Section 4, including the effective temperature approach capturing the effect of water uptake and degradation on the mechanical properties. Details on the numerical implementation of the model are presented in Section 5. Model predictions are compared to experimental data in Section 6, and numerical examples are presented in Section 7, before concluding.

## 2 Experiments

### 2.1 Experimental methods

#### 2.1.1 Material and specimen preparation

The PLA used in this study is the Ingeo™ 4060D from Natureworks LLC, which was supplied in pellet form. This polymer has a glass transition temperature of about 56 °C (Chen et al., 2023) and melting temperature in the range 150–180 °C (NatureWorks LLC, 2024). The raw material was first dried in an oven at 50 °C for over 48 h, and then moulded into plates at 180 °C in a vacuum oven. A pressure of 1 bar was applied in the vacuum oven to facilitate degassing and remove trapped air bubbles within the molten material. The moulding process was carried out for 3 h to obtain well-formed, bubble-free PLA plates. The PLA plates were polished on both sides and machined into specimen of desired shapes. Specimens for degradation studies consisted of long rods (length: 100mm, diameter: 8mm). This geometry was selected to obtain axisymmetric water uptake and degradation profiles that are approximately independent of the axial position along the rod. Thin discs (diameter: 10 mm, thickness: 1 mm) were also machined for water uptake measurements in the wet, undegraded state.

#### 2.1.2 Degradation tests

**Fig. 1:**
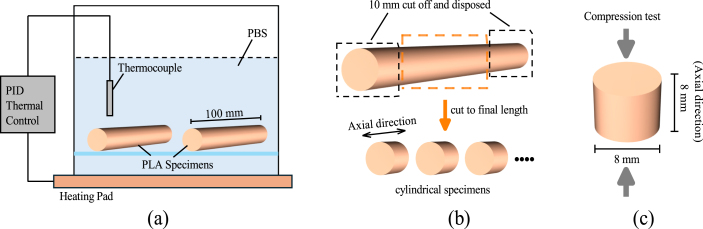
Schematic illustration of (a) the experimental setup for the degradation of PLA rods; (b) specimen preparation for mechanical testing; (c) compression testing on cylinder specimens.

Rod and disc specimens were immersed in phosphate buffered saline (PBS) solution (Sigma Aldrich, UK) with pH of 7.5 at 45 °C for up to 45 days (Fig. 1(a)). This temperature was selected to accelerate the degradation process. We have shown in our previous work that the degradation mechanism does not change in the range 37°–60 °C (Chen et al., 2026). The temperature was regulated using a PID thermal control system with a 0.2 °C tolerance, with real-time monitoring provided by two thermocouples. pH measurements were taken every two days and the PBS solution was replaced every 5 days.

Selected specimens were removed from the bath at predefined intervals, weighed and dried in a vacuum oven at room temperature for 4 days under a degassing pressure of 1 bar. The drying duration was selected to ensure that the mass does no longer change. The specimen masses were recorded in the dry state before degradation ($m_{d}\left(0\right)$), wet state after some period of degradation ($m_{w}\left(t\right)$), and dry state after degradation ($m_{d}\left(t\right)$). The water uptake ${\overset{¯}{\omega }}_{w}$ was calculated as ${\overset{¯}{\omega }}_{w}\left(t\right)=\frac{m_{w}\left(t\right)-m_{d}\left(t\right)}{m_{d}\left(t\right)}$, and the mass loss ratio $\overset{¯}{f}$ was calculated as $\overset{¯}{f}\left(t\right)=\frac{m_{d}\left(t\right)-m_{d}\left(0\right)}{m_{d}\left(0\right)}$. Here, the overbar indicates an average value over the sample volume. At least three mass measurements were taken for each condition. The diameters of wet specimens after various exposure times were also recorded.

The molecular weight of the dry, degraded rod specimens was measured using an HPLC gel permeation chromatography (GPC) system (Shimadzu, UK), calibrated using polystyrene standards. Polystyrene (PS) standards were used for calibration due to their commercial availability and well-characterised narrow molecular-weight distributions spanning a broad range of molecular weights, in the absence of comparable and readily available standards for PLA. Therefore, the molecular weights reported in this work should be understood as PS-equivalent molecular weights. To quantify degradation heterogeneity along the radial direction in rod specimens, samples (8 mg) were taken from the rod surface and rod centre using a scissor cutting technique. An average measure for the whole volume was also obtained by taking a circular sector from the cylinder. Samples were dissolved in tetrahydrofuran (THF) solvent at a concentration of 10 mg/ml and GPC analysis was conducted at an average flow rate of 1.0 mL/min at room temperature. For each condition, at least three repeat measurements were taken using different specimens. The distribution of molecular weight was recorded and the number average molecular weight ${\overset{¯}{M}}_{n}$ was calculated from the GPC curves (again, the overbar indicates an average over a sample volume).

#### 2.1.3 Mechanical testing

After a predefined degradation period, wet rods were cut into cylindrical specimens (diameter: 8 mm, length: 8 mm) for compression testing (Fig. 1b,c). Only specimens extracted far from the rod ends were used, so that properties could be assumed homogeneous in the axial direction. Uniaxial compression tests on dry undegraded and wet degraded specimens were performed using a commercial screw-driven load frame (Instron 5980) equipped with a 10 kN load cell and an environmental chamber. Prior to the compression tests, Vaseline${}^{\text{®}}$ jelly (Unilever, UK) was applied to the contact surfaces between the specimen and the compression platens to minimise friction and avoid barelling effects. In the few instances where barrelling was detected, results were discarded and tests were repeated. Compression tests on the dry undegraded specimens were performed at a constant true strain rate of 0.005 s^−1^, across various temperatures from 37 °C to 47 °C in 5 °C increments, and from 48 °C to 51 °C in 1 °C increment. Compression tests on the wet degraded samples were conducted at the same true strain rate at 37 °C. A humidifier was used to limit water evaporation during testing. Typically, two repeat tests were conducted per temperature for the undegraded specimens, and three to five repeats were conducted for the wet degraded specimens.

The average compression true stress was calculated as F/A, where F is the applied force and A the current cross-section area. In compression, the inelastic response of PLA is dominated by shear yielding (Chen et al., 2023), which is essentially volume preserving (Argon, 2013). We have verified this assumption by measuring the volume change in compression tests using DIC (results not shown here). Therefore, A was estimated as $A=A_{0}/\left(1+{ɛ}_{n}\right)$, where $A_{0}$ is the initial cross-section area and ${ɛ}_{n}$ is the nominal strain in the loading direction. Note that in calculating the current area, we have neglected the volume change due to elastic deformations (the Poisson’s ratio of PLA obtained from DIC measurements is 0.35 (Pan et al., 2024)). This approximation is acceptable considering that plastic deformations are much larger than elastic deformations. The compression true strain is defined by $-\ln\left(1+{ɛ}_{n}\right)$.

#### 2.1.4 DSC analysis

Differential scanning calorimetry (TA DSC Q2000) was employed to measure the glass transition temperature $T_{g}$. For dry degraded specimens, samples were taken from the middle of the rod, where the molecular weight was also measured. The glass transition temperature of the wet, undegraded polymer was measured using thin disc specimens immersed in water. Considering the fast uptake of water in thin specimens, we limited the absorption period to 2 days, which was sufficient to reach a saturation plateau, while the molecular weight decreased by only a few percents. A circular sector specimen, representative of the overall water content, was used for the DSC measurements. Hermetic pans were used to prevent water evaporation during testing of the wet samples. Samples were heated from 0 to 100 °C at a constant rate of 5 °C/min. Two measurements were performed on different specimens for each case. The glass transition temperature was determined by using a half-height technique in the transition region of the heat flow diagram.

### 2.2 Experimental results

#### 2.2.1 Degradation behaviour of PLA

**Fig. 2:**
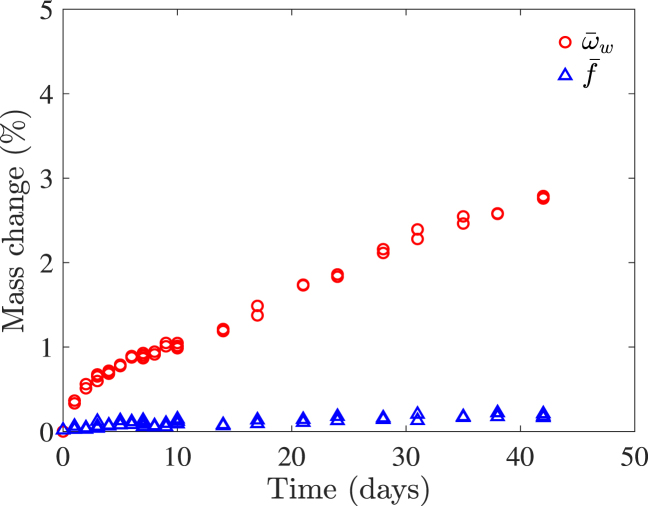
Average water uptake and mass loss as a function of time in rod specimens. All repeats are shown.

The evolution of average water uptake ${\overset{¯}{\omega }}_{w}$ and mass loss ratio $\overset{¯}{f}$ in PLA rods during degradation tests is shown in Fig. 2. The water content increased rapidly in the first 4 days to 0.7%, and then continued to rise steadily at an approximately constant rate. These results are consistent with our recent study (Chen et al., 2026) for the water uptake in PLA cylinders during degradation at 45 °C, with small differences attributed to the different geometry. The initial rapid uptake is attributed to diffusion of water into the specimen. In the absence of degradation, one would expect the water uptake to reach a plateau value, corresponding to the equilibrium water content in the polymer at that temperature. For example, a plateau at ${\overset{¯}{\omega }}_{w}=0.93$% was observed in hydration tests of the same PLA at 37 °C for up to 20 days, during which degradation was negligible (Chen et al., 2023). In contrast, no plateau was observed at 45 °C. This can be attributed to an earlier onset of hydrolytic degradation, as seen from the GPC results shown below. Hydrolytic chain scission increases the concentration of hydroxyl and carboxylic end groups, which are more hydrophilic than the ester bonds, increasing the equilibrium water content. In addition, no significant change in the specimen diameters was recorded, indicating negligible swelling within the considered time frame. Mass loss was also negligible during the studied degradation time, with $\overset{¯}{f}$ smaller than 0.2% after 45 days.

**Fig. 3:**
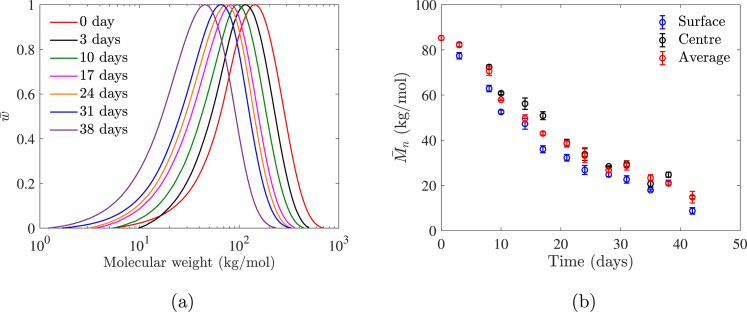
(a) Representative normalised molecular weight distributions in rod specimens (in the volume average sense) after different degradation periods. (b) Time evolution of number averaged molecular weights on the surface, centre and in an average sense in the cylinder samples.

Chain scission due to hydrolysis translates in a reduction in the chain molecular weight. Representative GPC curves corresponding to the volume-average measure in rod specimens are shown in Fig. 3(a) for various degradation periods. As degradation progressed, the molecular weight distribution shifted to the left while remaining unimodal. Similar trends were observed in the molecular weight distributions measured on the surface and in the centre of the degraded rods (not shown). The time evolution of the number-average molecular weight on the surface, centre, and in the volume-average sense is illustrated in Fig. 3(b). In the initial, undegraded state, the molecular weight was 85.7 kg/mol and decreased with exposure time in PBS. The surface degraded faster than the centre, suggesting that hydrolytic degradation is limited by the diffusion of water at this temperature and for the considered specimen geometry. At the end of the testing period, ${\overset{¯}{M}}_{n}$ reached approximately 10 kg/mol. This threshold value was identified in our recent work (Chen et al., 2026) as marking the onset of the ductile-to-brittle transition, which could be due to the molecular weight approaching the molecular weight between entanglements, estimated at 8.7 kg/mol for PLA (Dorgan et al., 1999).

#### 2.2.2 Mechanical behaviour of undegraded and wet degraded PLA

**Fig. 4:**
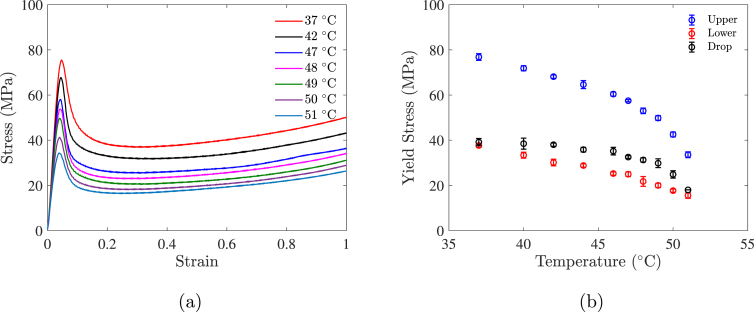
(a) Compressive true stress strain curves and (b) upper yield stress, lower yield stress, and yield stress drop of dry, undegraded PLA before at different temperatures.

Fig. 4(a) shows the compressive true stress–strain response of dry undegraded PLA at different temperatures. All curves show elastic deformation, yielding, significant softening, and slight hardening within the considered strain range. The upper yield stress (peak stress) and lower yield stress (lowest post-yield stress) are shown as a function of temperature in Fig. 4(b). The upper and lower yield stresses exhibit distinct temperature dependencies, resulting in the yield drop also depending on temperature. This mechanical response is not typical of all glassy polymers and is referred to as thermo-rheologically complex behaviour (Van Breemen et al., 2012). A slight decrease in the rehardening slope with increasing temperature can also be seen in Fig. 4(a).

**Fig. 5:**
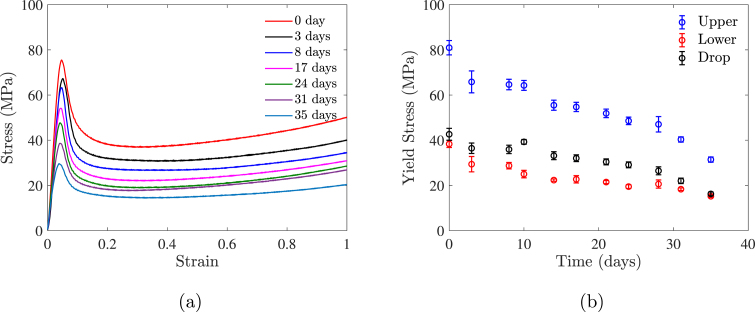
(a) Compressive true stress strain curves and (b) upper yield stress, lower yield stress, and yield stress drop of wet, degraded PLA after various degradation times.

Representative compressive stress–strain curves at 37 °C for degraded PLA cylinders in the wet state are shown in Fig. 5(a). The most noticeable effect of hydrolytic degradation is a reduction in the yield stress. The evolution of the upper yield stress, lower yield stress, and yield stress drop with degradation time is shown in Fig. 5(b). A pronounced and rapid drop in yield stress is observed at the early stage of degradation. This quick reduction is primarily attributed to water absorption by the polymer, which increases chain mobility and weakens intermolecular interactions, leading to an immediate loss of yield stress. Following this initial drop, the yield stress continued to decrease more gradually with increasing degradation time. This slower, long-term decline is associated with hydrolytic degradation-induced molecular weight reduction. We also note a faster degradation of the upper yield stress after about 25 days, compared to the lower yield stress. A reduction in the rehardening slope with degradation time can also be observed in Fig. 5(a). The similarity between the effect of increase in temperature (Fig. 4) and degradation time (Fig. 5) is evident. Note that we only show results for up to 35 degradation days, as experimental results became unreliable past this point due to the ductile-to-brittle transition. Specifically, up to 35 days, no cracks were observed during testing. At approximately 38 days, the cylindrical specimens were still able to sustain a certain level of load. However, multiple distributed radial cracks with crack surface parallel to the cylinder axis initiated at the outer surface and propagated towards the centre. After 45 days, the cylinders became highly brittle and fractured immediately into several irregularly shaped fragments under a very small load.

It is worth noting that, although the stress and strain are calculated using the same method as for undegraded samples, the stress–strain curves do not directly represent the material behaviour. Instead, they reflect the volume-averaged deformation behaviour, as the degradation status across the radius was not homogeneous.

#### 2.2.3 Glass transition temperature

**Fig. 6:**
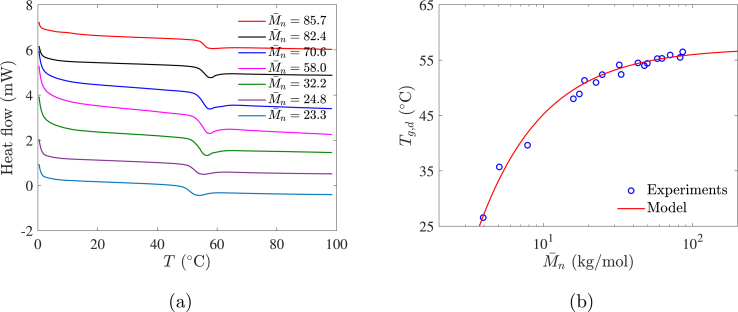
(a) Representative DSC curves for the dry degraded polymer at different degradation stages, labelled in terms of remaining number average molecular weight (units: kg/mol). (b) Evolution of the glass transition temperature extracted from the DSC curves with molecular weight.

We characterised the individual contributions of the reduction in molecular weight and water uptake on the evolution of the glass transition temperature measured by DSC analysis on thin disc specimens. Fig. 6(a) shows representative DSC curves for dry, degraded specimens after various degradation times. In the figure, curves are labelled by the corresponding number average molecular weight, measured by GPC. These curves confirm that the polymer was initially purely amorphous, and remained amorphous for most of the degradation period considered in this work. Degradation-induced crystallisation became only noticeable below ${\overset{¯}{M}}_{n}=10$ kg/mol. The evolution of the glass transition temperature calculated based on the DSC curves is shown as a function of ${\overset{¯}{M}}_{n}$ in Fig. 6(b). The glass transition temperature was 56.5 °C in the dry, undegraded polymer. The glass transition temperature then decreased as the molecular weight decreased, showing a large drop around ${\overset{¯}{M}}_{n}=10$ kg/mol. These results are consistent with data reported in Chen et al. (2026). The reduction in $T_{g}$ with a reduction in chain length has been attributed to the increasing number of chain ends, which disrupt molecular packing and facilitate cooperative motion of polymer segments (Wu and Buckley, 2004, Xu et al., 2025). The dependence of $T_{g}$ on the number average molecular weight can be described using the Flory-Fox equation (Fox and Flory, 1950):

(2) $$T_{g,d}=T_{g,\infty }-\frac{B}{M_{n}}$$

where we use the notation $T_{g,d}$ to indicate the glass transition temperature of the dry, degraded polymer. In Eq. (2), $T_{g,\infty }$ represents the theoretical glass transition temperature of the dry polymer with infinitely large molecular weight, and B is a fitting parameter. Predictions of the Flory-Fox equation (red line in Fig. 6(b)) are in excellent agreement with experimental data, taking $T_{g,\infty }=330.2$ K and B=120 K kg/mol.

**Fig. 7:**
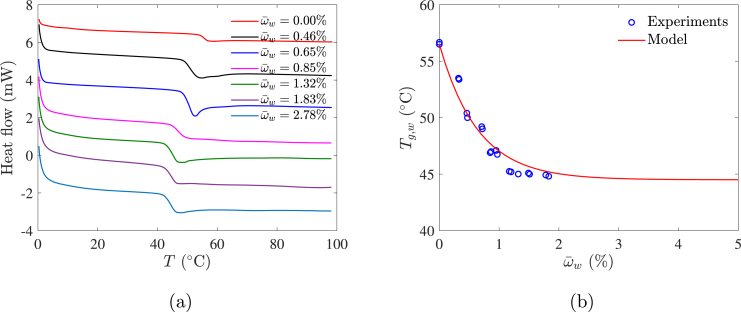
(a) Representative DSC curves of the wet undegraded polymer after different exposure times in water, labelled in terms of average water absorption ratio. (b) Evolution of the glass transition temperature extracted from the DSC curves with the water absorption ratio.

We next examine the role of water content on the reduction in $T_{g}$ in the undegraded PLA. Fig. 7(a) shows representative DSC curves for wet PLA obtained from the thin disc specimens immersed in water for up to 2 days. This duration was selected so that the thin discs could reach full saturation (corresponding to a plateau in the water uptake curves for thin discs), while degradation could be considered negligible based on GPC measurements. In the figure, curves are labelled based on the average water uptake in the whole specimen. The DSC heat-flow curves exhibit a systematic shift of the glass-transition region towards lower temperatures with increasing the water fraction, indicating enhanced chain mobility. The evolution of the glass transition temperature extracted from these curves is shown in Fig. 7(b). The glass transition temperature decreased from its initial value of 56.5 °C to 45.3 °C as the water uptake increased to ${\overset{¯}{\omega }}_{w}\approx 1$%. This reduction in $T_{g}$ is consistent with that reported in Chen et al. (2023) during hydration of the same PLA at 37 °C. The decrease of $T_{g}$ with the water uptake has been attributed to the plasticisation effect of water, which enhances the segmental mobility by disrupting intermolecular interactions (Siemann, 1985, Levine and Slade, 1988, Vyavahare et al., 2014). Beyond a water uptake of 1%, the glass transition temperature seemed to approach a plateau, which is attributed to the saturation in bound water when the total water fraction reaches a certain threshold. Note that the molecular weight decreases by less than 3kg/mol after two days of water absorption, corresponding to a reduction in the glass transition temperature of less than 0.5°C, which amounts to an error of approximately 4%. Therefore, the observed reduction in $T_{g}$ can be attributed entirely to water uptake.

To describe the effect of water fraction on the glass transition temperature in the wet, undegraded polymer, we use an equation proposed by (Reimschuessel, 1978):

(3) $$\Delta T_{g,w}={\left(\Delta T_{g,0}\right)}^{1-\frac{\omega_{w}}{\omega_{l}}}-\Delta T_{g,0}$$

where $\Delta T_{g,0}$ represents the maximum reduction in the glass transition temperature caused by water, and $\omega_{l}$ denotes the threshold water fraction beyond which a further increase in total water fraction no longer reduces the glass transition temperature. Predictions of Eq. (3) are shown in Fig. 7(b) (red line), taking $\Delta T_{g,0}=12$ K and $\omega_{l}=0.016$.

### 2.3 Implications for modelling

Based on the experimental observations reported in the previous section, we make the following assumptions for the constitutive modelling of glassy polymers degrading by hydrolysis:

• Water uptake is described using a reaction–diffusion equation, accounting for the increase in hydrophilicity brought about by degradation (Fig. 2). We neglect mass loss by the transport of short chains, since significant mass loss was not detected before the ductile-to-brittle transition.
• Since the water uptake remains small (Fig. 2), and no volume change could be measured in our experiments, we neglect swelling of the polymer by absorption of water.
• Heterogeneous degradation (Fig. 3(b)) suggests some degree of diffusion-limited hydrolysis. Therefore, the hydrolysis rate is taken as a function of the water concentration. The role of stresses on hydrolysis kinetics is neglected, since stresses were found to accelerate degradation only when the number average molecular weight fell below approximately 10 kg/mol for this PLA at 45 °C (Chen et al., 2026).
• We make an analogy between the effect of an increase in temperature and degradation (reduction in molecular weight and water uptake), and adopt the concept of effective temperature to describe the effect of degradation on the mechanical response, following Söntjens et al. (2012). The effective temperature is determined based on the reduction in $T_{g}$ with degradation.

## 3 Continuum thermodynamic framework

### 3.1 Kinematics

Let X denote the position of a material point in the initial, reference state. In the current state, the position of the material point is described by the one-to-one mapping x=x(X,t), and the material velocity is $v=\overset{˙}{x}$. The relative motion of material points is measured through the deformation gradient:

(4) $$F=\frac{\partial x}{\partial X}$$

and the Jacobian of the deformation gradient is defined as: J=det(F)>0. The deformation gradient is further decomposed multiplicatively as:

(5) $$F=F^{e}F^{p}$$

where $F^{e}$ and $F^{p}$ represent the elastic and plastic deformation gradient, respectively. As previously mentioned, we do not account for swelling due to water uptake, since we did not measure any swelling in our experiments.

### 3.2 Mechanical equilibrium

Consider an arbitrary volume of polymer subjected to a nominal surface traction T(X,t). Neglecting inertial effects and body forces, force balance requires that:

(6) ∇⋅P=0

where P is the first Piola–Kirchhoff stress tensor. On the other end, balance of moments requires that $PF^{T}=FP^{T}$. The first Piola–Kirchhoff stress P tensor and traction vector T are related by Cauchy’s relation:

(7) T=P⋅N

where N is the outward unit normal vector to a plane in the reference configuration. We also recall the usual relations between the Cauchy stress tensor and the first Piola–Kirchhoff stress tensor:

(8) $$P=J\sigma F^{-T},\;\sigma =J^{-1}PF^{T}$$

Our constitutive model for the mechanical response of the polymer is based on a conventional “glass-rubber” phenomenological framework, according to which the total stress is additively composed into two terms, respectively corresponding to an elasto-viscoplastic branch and a hyperelastic branch (Pan et al., 2024):

(9) $$P=P^{\left(1\right)}+P^{\left(2\right)},\;\sigma =\sigma^{\left(1\right)}+\sigma^{\left(2\right)}$$

where $P^{\left(1\right)}$, $\sigma^{\left(1\right)}$ and $P^{\left(2\right)}$, $\sigma^{\left(2\right)}$ are the stresses in the elasto-viscoplastic and hyperelastic branches, respectively. The elasto-viscoplastic branch describes intermolecular interactions in the polymer, controlling the initial elastic response, yielding, and post-yield softening leading to a plateau. The hyperelastic branch describes rehardening at large deformations, attributed to the stretching and alignment of polymer strands between entanglements. The additive decomposition (9) is motivated by the corresponding additive decomposition of the total mechanical free energy, which is discussed in Section 3.5.

### 3.3 Conservation of water and hydrolytic degradation

Let $C_{w}\left(X,t\right)$ be the nominal molar concentration of water (number of moles per unit volume in the reference configuration). The degradation state is described using an internal variable ξ(X,t) representing the extent of degradation (number of moles of chain scissions per unit volume in the reference configuration) (Pan and Brassart, 2023). Conservation of water molecules requires that:

(10) $${\overset{˙}{C}}_{w}=-\nabla \cdot J_{w}+R_{w}$$

where the notation $\overset{˙}{\left(\cdot \right)}$ represents the partial derivative with respect to time: $\overset{˙}{\left(\cdot \right)}\equiv \frac{\partial \left(\cdot \right)}{\partial t}\left(X,t\right)$. $\nabla \cdot J_{w}$ denotes the material divergence of the diffusion flux of water $J_{w}\left(X,t\right)$, and $R_{w}\left(X,t\right)$ is the production rate of water due to chain scission. Since each chain scission event consumes one water molecule, we simply have:

(11) $$R_{w}=-\overset{˙}{\xi }$$

Neglecting mass loss, the number average molecular weight can be related to the extent of reaction by:

(12) $$\frac{1}{M_{n}}=\frac{1}{M_{n,0}}+\frac{\xi }{\rho_{0}}$$

where $M_{n,0}$ denotes the initial average molecular weight and $\rho_{0}$ is the initial density of the polymer: $\rho_{0}=1.24\times 10^{3}\;{\mathrm{kg/m}}^{3}$.

### 3.4 Free energy imbalance

Consider a polymer body occupying volume B with surface ∂B in the reference configuration, and let ψ be the nominal free energy density of the degrading polymer (energy per unit volume in the reference configuration). Assuming a constant uniform temperature, thermodynamics requires that the external powers be larger than the rate of change in free energy of the polymer:

(13) $$\int_{\partial B}T\cdot v\;dA-\int_{\partial B}\mu_{w}J_{w}\cdot N\;dA-\frac{d}{dt}\int_{B}\psi \;dV\geq 0$$

where $\mu_{w}$ is the chemical potential of water (energy per mole). The first term represents the power of the external tractions and the second term represents the chemical power supplied by diffusion of water into the polymer. Inserting Cauchy’s relation (7) and using the divergence theorem, the mechanical power becomes:

(14) $$\int_{\partial B}T\cdot v\;dA=\int_{B}\left(\nabla \cdot P\right)\cdot v\;dV+\int_{B}P:\overset{˙}{F}\;dV$$

where the first term on the right hand side vanishes by virtue of the local equilibrium condition (6). Using the divergence theorem and the water conservation Eq. (10), the chemical power becomes:

(15) $$-\int_{\partial B}\mu_{w}J_{w}\cdot N\;dS=\int_{B}\left(\mu_{w}{\overset{˙}{C}}_{w}-\mu_{w}R_{w}-\nabla \mu_{w}\cdot J_{w}\right)dV$$

Inserting these relations into the inequality (13), and bringing the time derivative into the integral, we obtain the local inequality:

(16) $$P:\overset{˙}{F}+\mu_{w}{\overset{˙}{C}}_{w}+\mu_{w}\overset{˙}{\xi }-J_{w}\cdot \nabla \mu_{w}-\overset{˙}{\psi }\geq 0$$

where we also used Eq. (11).

Inserting the stress decomposition (9) and the multiplicative decomposition of the deformation gradient (5), the stress power can be re-expressed as:

(17) $$P:\overset{˙}{F}=P^{e\left(1\right)}:{\overset{˙}{F}}^{e}+M^{\left(1\right)}:L^{p}+P^{\left(2\right)}:\overset{˙}{F}$$

where the stress-like tensors $P^{e\left(1\right)}$ and $M^{\left(1\right)}$ are defined as:

(18) $$P^{e\left(1\right)}=P^{\left(1\right)}{F^{p}}^{T}$$

(19) $$M^{\left(1\right)}={F^{e}}^{T}P^{\left(1\right)}{F^{p}}^{T}$$

and $L^{p}$ is the plastic spatial velocity gradient:

(20) $$L^{p}={\overset{˙}{F}}^{p}{F^{p}}^{-1}$$

Using Eq. (17), the local inequality (16) becomes:

(21) $$P^{e\left(1\right)}:{\overset{˙}{F}}^{e}+M^{\left(1\right)}:L^{p}+P^{\left(2\right)}:\overset{˙}{F}+\mu_{w}{\overset{˙}{C}}_{w}+\mu_{w}\overset{˙}{\xi }-J_{w}\cdot \nabla \mu_{w}-\overset{˙}{\psi }\geq 0$$

### 3.5 State laws and reduced inequality

We assume that the free energy can be additively decomposed into mechanical, mixing and reaction contributions (Konica and Sain, 2020, Pan and Brassart, 2022):

(22) $$\psi \left(F,F^{e},C_{w},\xi \right)=\psi^{mech}\left(F,F^{e},C_{w},\xi \right)+\psi^{mix}\left(C_{w},\xi \right)+\psi^{reac}\left(\xi \right)$$

The mechanical contribution to the free energy is further decomposed into two contributions (Pan et al., 2024):

(23) $$\psi^{mech}\left(F,F^{e},C_{w},\xi \right)=\psi^{\left(1\right)}\left(F^{e},C_{w},\xi \right)+\psi^{\left(2\right)}\left(F,C_{w},\xi \right)$$

where $\psi^{\left(1\right)}$ and $\psi^{\left(2\right)}$ denote the free energy of each branch. The free energy of Branch 1 captures the energetic costs associated with stretching intermolecular bonds between the polymer chains. The free energy of Branch 2 represents the energetic cost associated with stretching and alignment of the polymer strands between entanglements. Since the two molecular mechanisms coexist at the molecular scale, simple additivity of the corresponding free energies is assumed without any weighting factor, while the density of bonds or chains involved in each process is lumped into the elastic moduli parameterising these free energies and introduced in the next section. Inserting the specific forms (22), (23) into the inequality (21), we find:

(24) $$\left(P^{e\left(1\right)}-\frac{\partial \psi^{\left(1\right)}}{\partial F^{e}}\right):{\overset{˙}{F}}^{e}+\left(P^{\left(2\right)}-\frac{\partial \psi^{\left(2\right)}}{\partial F}\right):\overset{˙}{F}+M^{\left(1\right)}:L^{p}+\left(\mu_{w}-\frac{\partial \psi }{\partial C_{w}}\right){\overset{˙}{C}}_{w}+\left(\mu_{w}-\frac{\partial \psi }{\partial \xi }\right)\overset{˙}{\xi }-J_{w}\cdot \nabla \mu_{w}\geq 0$$

This expression identifies the driving forces associated with elastic and plastic deformation, insertion of water, chemical degradation and water transport. Neglecting viscoelastic deformations, we obtain the state laws for the stress in each branch:

(25) $$P^{e\left(1\right)}=\frac{\partial \psi^{\left(1\right)}}{\partial F^{e}},\;P^{\left(2\right)}=\frac{\partial \psi^{\left(2\right)}}{\partial F}$$

The corresponding Cauchy stresses are obtained from Eq. (8):

(26) $$\sigma^{\left(1\right)}=\frac{1}{J}\frac{\partial \psi^{\left(1\right)}}{\partial F^{e}}{F^{p}}^{T},\;\sigma^{\left(2\right)}=\frac{1}{J}\frac{\partial \psi^{\left(2\right)}}{\partial F}F^{T}$$

We also assume equilibrium w.r.t. to the insertion of water, giving the state law for the chemical potential of water:

(27) $$\mu_{w}=\frac{\partial \psi }{\partial C_{w}}=\frac{\partial \psi^{mech}}{\partial C_{w}}+\frac{\partial \psi^{mix}}{\partial C_{w}}$$

Using the state laws (25), (27), the free energy inequality (24) reduces to:

(28) $$M^{\left(1\right)}:{\overset{˙}{L}}^{p}+A\overset{˙}{\xi }-J_{w}\cdot \nabla \mu_{w}\geq 0$$

where A is the chemical affinity driving chemical degradation:

(29) $$A\equiv \mu_{w}-\frac{\partial \psi }{\partial \xi }=\mu_{w}-\frac{\partial \psi^{mech}}{\partial \xi }-\frac{\partial \psi^{mix}}{\partial \xi }-\frac{\partial \psi^{reac}}{\partial \xi }$$

In classical chemical thermodynamics (Kondepudi and Prigogine, 2014), the chemical affinity is the difference between the chemical potentials of the products and the reactants, weighted by their stoichiometric coefficients. Here, since only water was considered as mobile species, only the chemical potential of water explicitly appears in the expression of the affinity. The chemical potentials of the other reactant (the ester bonds) and products (the chain ends) are accounted for in an implicit manner through the reaction contribution $\psi^{reac}$ to the free energy. The affinity also accounts naturally for the changes in mechanical and mixing free energy arising from degradation.

The inequality should be satisfied by the kinetic models of viscoplastic flow, chemical reaction and water diffusion. Assuming that these processes can occur independently, we require that:

(30) $$M^{\left(1\right)}:{\overset{˙}{L}}^{p}\geq 0,\;A\overset{˙}{\xi }\geq 0,\;-J_{w}\cdot \nabla \mu_{w}\geq 0$$

Specific forms for the state laws and kinetic models are presented in the next section.

## 4 Specific constitutive models

### 4.1 Elasto-viscoplastic deformation

#### 4.1.1 Free energy

The strain energy in the elasto-viscoplastic branch is expressed using the quadratic Hencky model based on the elastic logarithmic strain:

(31) $$\psi^{\left(1\right)}=\mu^{\left(1\right)}\text{tr}\left(E^{e2}\right)+\frac{1}{2}\left(\kappa^{\left(1\right)}-\frac{2}{3}\mu^{\left(1\right)}\right){\left(\text{tr}E^{e2}\right)}^{2}$$

where $\mu^{\left(1\right)}=\frac{E^{\left(1\right)}}{2\left(1+\nu^{\left(1\right)}\right)}$ and $\kappa^{\left(1\right)}=\frac{E^{\left(1\right)}}{3\left(1-2\nu^{\left(1\right)}\right)}$ are respectively the shear and bulk moduli in the elasto-viscoplastic branch, with $E^{\left(1\right)}$ the corresponding Young’s modulus and $\nu^{\left(1\right)}$ the Poisson’s ratio. $E^{e}=\text{diag}\left\{E_{1}^{e},E_{2}^{e},E_{3}^{e}\right\}$ in the principal basis, where $E_{i}^{e}=\text{ln}\left(\lambda_{i}^{e}\right)$ (i=1,2,3) are the principal elastic logarithmic strains, and $\lambda_{i}^{e}$ are the principal elastic stretches. The compressibility of this branch accounts for the elastic compressibility of the polymer, recalling that the Poisson’s ratio of PLA is 0.35. The Hencky strain energy (31) has been used in previous works addressing the large deformation behaviour of polymers (Anand, 1979, Srivastava et al., 2010). While this model has some known mathematical deficiencies and can lead to unphysical behaviour at very large deformation (Neff et al., 2015), it should be noted that elastic strains remain small in our problem, as large deformation can be accommodated by plastic strain.

The strain energy in Branch 2 is described using the compressible Gent model (Horgan and Saccomandi, 2004):

(32) $$\psi^{\left(2\right)}=-\frac{\mu^{\left(2\right)}}{2}J_{m}\;\text{ln}\left(1-\frac{I_{1}-3}{J_{m}}\right)+\frac{\kappa^{\left(2\right)}}{2}{\left(\text{ln}J\right)}^{2}$$

where $\mu^{\left(2\right)}=\frac{E^{\left(2\right)}}{2\left(1+\nu^{\left(2\right)}\right)}$ and $\kappa^{\left(2\right)}=\frac{E^{\left(2\right)}}{3\left(1-2\nu^{\left(2\right)}\right)}$ are respectively the shear and bulk moduli of Branch 2, with $E^{\left(2\right)}$ the Young’s modulus and $\nu^{\left(2\right)}$ the Poisson’s ratio in the hyperelastic branch; $I_{1}$ is the first invariant of the deformation and $J_{m}$ is a material parameter controlling the maximum extensibility. A small compressibility was used in the hyperelastic branch to provide a realistic description of the hydrostatic response and for numerical stability. A similar approach has been adopted in other studies (Sain et al., 2018, Bergström, 2015).

The corresponding Cauchy stresses are obtained from Eq. (26) as:

(33) $$\sigma^{\left(1\right)}=\frac{1}{J}\left[2\mu^{\left(1\right)}\left(\text{dev}E^{e}\right)+\kappa^{\left(1\right)}\left(\text{tr}E^{e}\right)1\right]$$

(34) $$\sigma^{\left(2\right)}=\frac{1}{J}\left[\frac{\mu^{\left(2\right)}J_{m}}{J_{m}-\left(I_{1}-3\right)}b+\kappa^{\left(2\right)}\left(\text{ln}J\right)1\right]$$

where $b=FF^{T}$ is the left Cauchy–Green tensor.

#### 4.1.2 Viscoplastic flow rule

The plastic dissipation in Eq. (30) can be equivalently rewritten as (Reese and Govindjee, 1998, Doghri, 2000, Pan et al., 2024):

(35) $$-\tau^{\left(1\right)}:\frac{1}{2}\left(L_{v}b^{e}\right){b^{e}}^{-1}\geq 0$$

where $\tau^{\left(1\right)}=J\sigma^{\left(1\right)}$ is the Kirchhoff stress in the viscoplastic branch, $b^{e}$ is the elastic left Cauchy–Green tensor: $b^{e}=F^{e}F^{eT}$, and $L_{v}b^{e}$ denotes the Lie derivative of $b^{e}$:

(36) $$L_{v}b^{e}=F\left(\frac{D\left(F^{-1}b^{e}F^{-T}\right)}{Dt}\right)F^{T}=\left(F{\overset{˙}{C}}^{p-1}F^{T}\right)$$

corresponding to the push forward of the rate of plastic deformation tensor ${\overset{˙}{C}}^{p-1}$ to the current configuration with $C^{p}=F^{pT}F^{p}$ (Simo and Hughes, 2006, Pan et al., 2024). In deriving (35), we have assumed that the plastic flow is irrotational, see Pan et al. (2024) for details. We assume that viscoplastic flow is governed by shear yielding, which can be considered as volume preserving. Therefore, we adopt a von Mises-based flow rule (Simo and Hughes, 2006):

(37) $$-\frac{1}{2}\left(L_{v}b^{e}\right)b^{e-1}=\overset{˙}{\gamma }N$$

where $\overset{˙}{\gamma }$ denotes the equivalent plastic strain rate and N specifies the flow direction and is given by:

(38) $$N=\frac{{\overset{\sim }{\tau }}^{\left(1\right)}}{\sqrt{2}\tau^{\left(1\right)}},\;\tau^{\left(1\right)}=\sqrt{\frac{1}{2}{\overset{\sim }{\tau }}^{\left(1\right)}:{\overset{\sim }{\tau }}^{\left(1\right)}}$$

where ${\overset{\sim }{\tau }}^{\left(1\right)}=\text{dev}\;\tau^{\left(1\right)}$ denotes the deviatoric part of $\tau^{\left(1\right)}$, so that tr(N)=0. We describe the stress-activated flow rate using an Eyring-type equation (Eyring, 1936, Govaert et al., 2000, Wismans et al., 2024):

(39) $$\overset{˙}{\gamma }={\overset{˙}{\gamma }}_{0}\text{exp}\left(-\frac{\Delta G}{k_{B}\overset{ˆ}{T}}+H\right)\text{sinh}\left(\frac{\tau^{\left(1\right)}V}{k_{B}\overset{ˆ}{T}}\right)$$

where ${\overset{˙}{\gamma }}_{0}$ is a reference flow rate, ΔG is the activation energy, V is the activation volume for shear yielding, and $k_{B}$ is the Boltzmann constant. Here, $\overset{ˆ}{T}=\overset{ˆ}{T}\left(C_{w},\xi \right)$ is the effective temperature, describing the effect of water uptake and chain scission on yielding and such that $\overset{ˆ}{T}=T$ (the testing temperature) in the dry, undegraded state. The expression of the effective temperature will be provided in the next section. Post-yield softening is described through the variable H, evolving with γ as (Boyce et al., 1988):

(40) $$\overset{˙}{H}=h\left(1-\frac{H}{H^{s}}\right)\overset{˙}{\gamma },\;H\left(0\right)=0$$

where h and $H^{s}$ are softening parameters related to the softening slope and steady-state yield strength, respectively. Noting that $\overset{˙}{\gamma }\geq 0$, and inserting the flow rule (37) together with Eq. (38) in the inequality (35), it is readily verified that the viscoplastic dissipation rate is non-negative.

In formulating our viscoplastic flow rule, we have assumed volume-preserving plastic flow, which is a reasonable assumption for inelastic deformation dominated by shear-yielding (Argon, 2013). However, as shown in our previous work (Chen et al., 2023), PLA can experience significant dilatational plastic deformations under tension due to crazing. In general, dilatational plastic deformations in polymers may also result from void formation or particle debonding, and depend on the triaxiality ratio (Ognedal et al., 2014). In Pan et al. (2024), we developed a constitutive model for PLA where a volume-preserving or dilatational flow rule is used depending on the local value of the mean stress. In principle, this model could be adopted to describe the behaviour of degrading PLA using the effective temperature approach, which would extend the range of applicability of the model to more general loading conditions. However, since we only have experimental data for the compression behaviour, we limited the model formulation to shear yielding in the present work. The Eyring-type flow rule for shear plasticity (39) could also be extended to account for pressure dependence, see e.g. Buckley and Jones (1995).

#### 4.1.3 Temperature-dependence of mechanical properties

We use the following equation to describe the temperature-dependence of the elastic moduli (Mahieux and Reifsnider, 2001):

(41) $$E^{\left(i\right)}=E_{0}^{\left(i\right)}\exp\left[-{\left(\frac{\overset{ˆ}{T}}{T_{E}}\right)}^{\beta_{E}}\right],\;i=1,2$$

where $E_{0}^{\left(i\right)}$ is the Young’s modulus in branch i at $\overset{ˆ}{T}=0$ K; $T_{E}$ denotes a reference temperature, and $\beta_{E}$ is a fitting constant. The parameters $\beta_{E}$ and $T_{E}$ are taken identical in both branches. The dependence of $E^{\left(1\right)}$ and $E^{\left(2\right)}$ on the effective temperature aims to capture the increase in chain mobility brought about by an increase in temperature or degradation, impacting the initial modulus of the polymer as well as its rehardening capacity (Govaert and Tervoort, 2004).

Our experimental results show that PLA exhibits a thermo-rheologically complex response, characterised by a temperature dependence of the yield drop (Fig. 4(b)). A similar response is observed in the dependence of the yield drop with degradation time (Fig. 5(b)). Using the flow rule (39) with constant ΔG and the softening function (40) with constant $H^{s}$ cannot capture this complex behaviour. Therefore, we assume that both ΔG and $H^{s}$ depend on the effective temperature:

(42) $$\Delta G=\Delta G_{0}\exp\left[-{\left(\frac{\overset{ˆ}{T}}{T_{\Delta G}}\right)}^{\beta_{\Delta G}}\right],\;H^{s}=H_{0}^{s}\exp\left[-{\left(\frac{\overset{ˆ}{T}}{T_{H}}\right)}^{\beta_{H}}\right]$$

where $\Delta G_{0}$, $H_{0}^{s}$, $T_{\Delta G}$, $T_{H}$, $\beta_{\Delta_{G}}$ and $\beta_{H}$ are fitting parameters. Note that similar phenomenological functions have been used in Srivastava et al. (2010) and Lan et al. (2022) to describe the temperature-dependent properties of glassy polymers. Note that the dependence of these mechanical properties on water content and extent of degradation via $\overset{ˆ}{T}$ does not impact the dissipation inequality for viscoplastic flow.

In the literature, thermo-rheologically complex behaviour has been interpreted as evidence of multiple molecular processes contributing to yielding in glassy polymers, which can be captured by considering multiple viscoplastic branches in the constitutive model (Van Breemen et al., 2012). In principle, such a multi-branch description of yielding could also be adopted within the effective temperature framework. However, here we have retained a single viscoplastic branch but allowed the parameters ΔG and H to depend on the (effective) temperature in order to capture this behaviour while keeping the constitutive structure compact.

#### 4.1.4 Effective temperature

In order to express the effective temperature $\overset{ˆ}{T}$ as a function of the water concentration $C_{w}$ and extent of degradation ξ, we extend the approach proposed by Söntjens et al. (2012) and assume that the temperature difference $\overset{ˆ}{T}-T$ is proportional to the drop in glass transition temperatures between the degraded wet polymer ($T_{g}$) and intact dry polymer $T_{g,d0}$:

(43) $$\overset{ˆ}{T}\left(C_{w},\xi \right)=T+\eta \left(T_{g,d0}-T_{g}\left(C_{w},\xi \right)\right)$$

where η is a fitting parameter. It then remains to express the evolution of $T_{g}$ as a function of ξ and $C_{w}$. For simplicity, we will assume that the effects of average molecular weight and water content on the reduction in glass transition temperature are additive. Building on Eqs. (2), (3), we propose the following expression:

(44) $$T_{g}\left(C_{w},\xi \right)={\left(\Delta T_{g,0}\right)}^{1-\frac{\omega_{w}}{\omega_{l}}}-\Delta T_{g,0}+T_{g,d}\left(M_{n}\right)$$

with $T_{g,d}\left(M_{n}\right)$ given by the Flory-Fox Eq. (2) and $M_{n}$ is related to ξ by Eq. (12). The water fraction $\omega_{w}$ is related to the nominal water concentration $C_{w}$ by: $\omega_{w}=\frac{C_{w}M_{w}}{\rho_{0}}$, in which $M_{w}=18$ g mol^−1^ denotes the molar mass of water. We can verify that $T_{g,d0}$ is recovered with ω=0 and $M_{n}=M_{n,0}$ in the above equation. Note that all parameters in Eq. (44) have already been identified in Section 2.

### 4.2 Water transport and chemical degradation

#### 4.2.1 Water uptake

To describe mixing of polymer and water, we adopt the classical Flory–Huggins model (Flory, 1942, Huggins, 1942), accounting for entropic and enthalpic effects:

(45) $$\psi^{mix}\left(C_{w},\xi \right)=\mu_{w,0}C_{w}+\frac{RT}{v}\frac{1}{\left(1-\phi_{w}\right)}\left[\phi_{w}\ln\phi_{w}+\chi \left(\xi \right)\phi_{w}\left(1-\phi_{w}\right)\right]$$

where $\mu_{w,0}$ is a reference chemical potential corresponding to pure water, $\phi_{w}=\frac{vC_{w}}{1+vC_{w}}$ is the volume fraction of water, in which $v=1.8\times 10^{-5}\;m^{3}/\mathrm{mol}$ is the molar volume of water, χ is the interaction parameter between water and polymer chains, and R is the ideal gas constant. The chemical potential of water is then obtained from the state law (27) as (Chester and Anand, 2010, Pan and Brassart, 2022):

(46) $$\mu_{w}=\mu_{w,0}+RT\left[\ln\phi_{w}+\left(1-\phi_{w}\right)+\chi {\left(1-\phi_{w}\right)}^{2}\right]$$

Note that in writing Eq. (46), we have neglected the contribution $\frac{\partial \psi^{mech}}{\partial C_{w}}$ to the state law for the chemical potential (27). We have verified that the contribution to chemical potential due to the change in mechanical properties with water uptake is indeed negligible compared to the mixing contribution.

During degradation, hydroxyl and carboxylic acid end groups are created, which causes an increase in hydrophilicity and thus a decrease in χ (Sharp et al., 2001). While the increase in concentration of chain ends can be quantified via the extent of degradation ξ, the precise relationship between ξ and the interaction parameter is not known. Therefore, the following empirical expression is adopted:

(47) $$\chi =\chi_{0}\left(1-\alpha {\overset{¯}{\xi }}^{\beta }\right)$$

in which $\chi_{0}$ is the interaction parameter between the polymer and water in the initial state and α and β are constant fitting parameters. The normalised extent of reaction is defined as $\overset{¯}{\xi }=\frac{\xi }{C_{b,0}}$ where $C_{b,0}$ is the initial concentration of ester bonds, so that $\overset{¯}{\xi }$ ranges from 0 (initial undegraded state) to 1 (fully degraded state). The proposed form has several desirable properties: (i) it recovers the initial interaction parameter $\chi_{0}$ when ξ=0; (ii) it ensures a monotonic decrease of χ as degradation proceeds; and (iii) the parameters α and β provide sufficient flexibility to represent different degradation sensitivities while keeping the model compact.

#### 4.2.2 Kinetics of hydrolysis

The rate of chain scission is assumed to be proportional to the concentrations of water and ester bonds:

(48) $$\overset{˙}{\xi }=KC_{w}C_{b}\left(\xi \right)$$

where K is the rate constant which in general depends on temperature, and $C_{b}$ denotes the current concentration of ester bonds, which is related to the initial concentration of ester bonds $C_{b,0}$ by:

(49) $$C_{b}\left(\xi \right)=C_{b,0}-\xi$$

Eq. (48) constitutes a second-order reaction kinetic model, which neglects the reverse (condensation) reaction. The dependence of the degradation rate on water concentration is motivated by our experimental observations that degradation is faster on the surface, resulting in a faster reduction in molecular weight (Fig. 3(b)) and larger pores near the surface at advanced degradation stages (Chen et al., 2026). These observations suggest a certain degree of diffusion-limited degradation. The concentration of ester bonds can also be related to the number average molecular weight by:

(50) $$C_{b}=\left(\frac{1}{M_{1}}-\frac{1}{M_{n}}\right)\rho_{0}$$

where $M_{1}$ denotes the molar mass of one monomer. Considering that $M_{n}$ is always much larger than $M_{1}$ in this study (the final $M_{n}$ value reached in our experiments is more than one hundred times larger than $M_{1}$), Eq. (50) shows that $C_{b}$ remains approximately constant during the considered degradation period. Therefore, we neglect the change in $C_{b}$ in the reaction kinetic model (48) and set $C_{b}\approx C_{b0}$. For PLA, $M_{1}=72$ g/mol, and we find $C_{b,0}=1.72$ × $10^{4}$ mol/m${}^{3}$.

Compliance with the dissipation inequality (30) can be verified by expressing the kinetic model in the form (Prigogine et al., 1948):

(51) $$\overset{˙}{\xi }=KC_{w}C_{b}\left(\xi \right)\left(1-\exp\left(-\frac{A}{RT}\right)\right)$$

ensuring that the chemical affinity and reaction rate have always the same sign. Eq. (48) can be regarded as the limit of the more rigorous model (51) when the affinity is “large”. In principle, it is possible to postulate a free energy of reaction $\psi^{reac}\left(\xi \right)$ such that this limiting behaviour holds in the degradation range considered in this work. Ideally, the expression of the reaction free energy should be derived from first principles. However, the rigorous derivation of such an expression goes beyond the scope of this work and the simpler expression (48) is used.

The reaction kinetic model (48) (or alternatively (51)) could be generalised to account for the autocatalytic effect of the carboxylic end groups, by making the reaction constant K a function of the catalyst concentration (Lyu et al., 2007, Wang et al., 2008, Pan and Brassart, 2023). However, here we did not include autocatalysis as no clear evidence of it was observed experimentally for PLA under the considered degradation conditions. Moreover, introducing autocatalysis would mainly accelerate the overall degradation kinetics and is not expected to significantly improve the predictive accuracy of the current model. The kinetic model could also be generalised by taking the reaction constant to depend on the stress state. For example, Eyring-type expressions of the reaction kinetic constants have been used in the literature to describe stress-accelerated hydrolytic degradation (Li et al., 2017, Zanetti Ferreira et al., 2026). However, as previously mentioned, our experiments did now show evidence of stress-accelerated hydrolysis at the considered molecular weight values, and the precise mechanisms underlying the effects of stress on degradation remain poorly understood. Therefore, we did not account for stress-enhanced hydrolysis in this work.

#### 4.2.3 Diffusion of water

The kinetics of water transport by diffusion is described using Fick’s first law, which is a large-deformation setting and expressed in the reference configuration takes the following form (Hong et al., 2008, Brassart et al., 2018):

(52) $$J_{w}=-\frac{D_{w}C_{w}}{RT}\left(F^{-1}F^{-T}\right)\nabla \mu_{w}$$

where $D_{w}$ is the diffusion coefficient of water in the polymer. This kinetic model obviously satisfies the dissipation inequality (30) for diffusion.

## 5 Numerical implementation

The reaction–diffusion model was implemented as a user-defined thermal material subroutine (UMATHT) in Abaqus by making an analogy between reaction–diffusion and heat transfer. Since UMATHT requires all quantities to be formulated in the current configuration, we first rewrite the water conservation Eq. (10) in the current configuration:

(53) $$\frac{1}{J}\overset{˙}{\bar{Jc_{w}}}=-\mathrm{div}j_{w}+r_{w}$$

where $c_{w}=\frac{C_{w}}{J}$, $j_{w}=\frac{1}{J}FJ_{w}$, and $r_{w}=\frac{R_{w}}{J}$ are the true concentration of water, the true water flux, and the true water production rate, respectively. div(⋅) represents the spatial divergence operator. The true flux corresponding to Eq. (52) is

(54) $$j_{w}=-\frac{D_{w}c_{w}}{RT}\mathrm{grad}\mu_{w}$$

where grad(⋅) denotes the spatial gradient operator. Inserting Eq. (46) into Eq. (54) yields the true flux $j_{w}$ expressed in terms of the spatial gradient of water concentration:

(55) $$j_{w}=-{\overset{¯}{D}}_{w}\mathrm{grad}c_{w},\;{\overset{¯}{D}}_{w}=D_{w}\left(1-2\chi \phi_{w}\right){\left(1-\phi_{w}\right)}^{2}$$

where ${\overset{¯}{D}}_{w}$ represents the effective diffusion coefficient of water. In the reaction–diffusion and heat transfer analogy, the temperature field is replaced by the water concentration field, the heat flux is replaced by the water flux, the thermal conductivity is replaced by the effective water diffusion coefficient, the volumetric heat capacity is replaced by a unit storage coefficient, and the internal heat generation term is replaced by the reaction sink term associated with hydrolysis. Note that in our numerical implementation of the reaction–diffusion model, we neglected the change of volume and set J=1 for simplicity, which introduces a negligible error given the small volume change. The calculated distributions of water concentration and average molecular weight at a given time were then used as input for the mechanical simulation by storing them as state variables in the user subroutine (UMAT), which calculates the Cauchy stress. We solved the mechanical constitutive equations using an exponential mapping in combination with a classical elastic-predictor/plastic-corrector integration algorithm (Simo and Hughes, 2006, Pan et al., 2024). The return-mapping equations were further reduced into one scalar equation, which was solved using a fully implicit algorithm with the Newton–Raphson method. To ensure objectivity and fast convergence, the consistent Jacobian moduli (DDSDDE in Abaqus) were derived based on the Jaumann rate of the Kirchhoff stress tensor. Details about the numerical algorithm implemented in the UMAT can be found in Pan et al. (2024).

In the following numerical examples, two scenarios are successively considered: sequential simulations, and coupled simulations. In a sequential analysis, degradation occurs under no applied loads, and mechanical loads are applied on the degraded polymer. In this scenario, the 8-node linear brick diffusive heat transfer or mass diffusion element (DC3D8) was used for the degradation analysis, and the 8-node linear brick continuum element (C3D8) was used for the subsequent mechanical analysis. In coupled simulations, mechanical loads are applied while degradation takes place. In this case, we solve the reaction–diffusion and mechanical problems simultaneously by combining UMAT and UMATHT, and using the 8-node trilinear coupled temperature–displacement element (C3D8T). Note that only weak coupling is considered in this work, in which fields of water concentration and extent of degradation impact the mechanical properties, while mechanical fields do not impact the chemical fields.

## 6 Comparison to experiments

In this section, we use the model described in Section 4 to reproduce experimental data presented in Section 2.2. We first consider the degradation response (water uptake and reduction in molecular weight), independent of mechanics. Then, the viscoplastic model with temperature-dependent properties is calibrated on stress–strain curves for the dry, undegraded polymer tested at different temperatures. Finally, the effective temperature approach is used to model the response of the wet degraded polymer.

### 6.1 Hydrolytic degradation of PLA rods

**Fig. 8:**
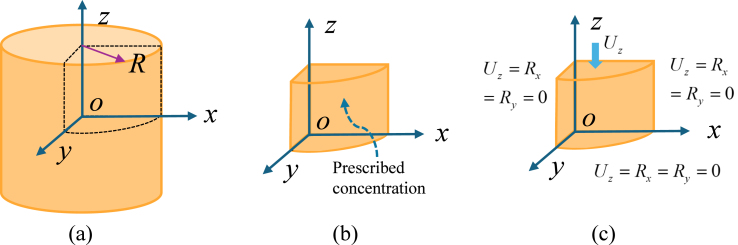
(a) Illustration of one-eighth of the cylindrical specimen, (b) boundary conditions for degradation modelling, and (c) boundary conditions for mechanical modelling.

We first consider the reaction and diffusion in a cylindrical rod. Given the large aspect ratio (12.5) of the rods used in the experiments, we assume homogeneous degradation in the axial direction, and only account for heterogeneous degradation along the radial direction. Although the problem can be simplified to a one-dimensional one, we still conducted three-dimensional finite element simulations in view of subsequent mechanical analysis (we however verified our finite element results against results obtained using a one-dimensional finite difference method with explicit time integration). Exploiting the symmetry of the problem, as shown in Fig. 8a, one-eighth of the cylindrical geometry was modelled and discretised using 384 DC3D8 elements. A prescribed water concentration was imposed on the cylindrical surface (Fig. 8b). The surface concentration was dynamically updated following Eqs. (46), (47), under the assumption that the outer surface remains in thermodynamic equilibrium with the water bath. The parameters related to water diffusion and hydrolytic reaction were adjusted to obtain the best fit with the experimental data and are listed in Table 1.

**Fig. 9:**
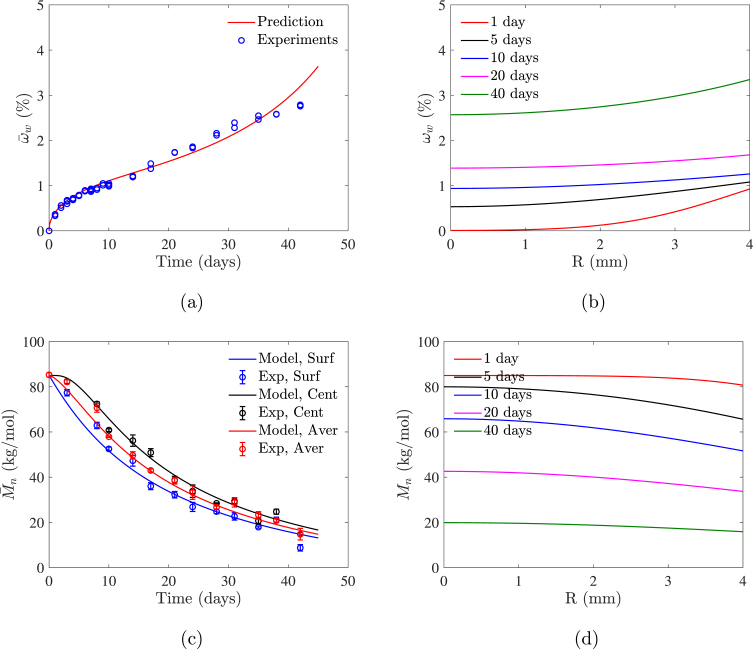
Model predictions for (a) the average water uptake, (b) the water uptake profile along the rod radius, (c) the average number average molecular weight, and (d) the number average molecular weight profile along the rod radius.

Fig. 9(a) compares model predictions for the average water uptake as a function of time to experimental data. A fairly good agreement was obtained over the considered degradation period, showing a transition from a diffusion-limited regime in the first days of exposure to a degradation-limited regime, where water uptake continued to increase due to the increase in affinity between polymer and water. Predicted profiles of water uptake along the radius R are shown in Fig. 9(b), showing gradual homogenisation of the water fraction profile with degradation time over the first 20 days of degradation as a result of diffusion-driven smoothing of spatial gradients. Beyond this period, the concentration gradient increased again, which is attributed to the increase in water content on the outer surface resulting from increased water affinity. The volume-averaged number average molecular weight predicted by the model is shown in Fig. 9(c), together with local predictions on the surface and at the centre, showing a very good agreement with the experimental data. In particular, the model captures the faster degradation on the surface, due to a higher concentration of water on the surface as shown in Fig. 9(b), and thus a higher reaction rate according to the kinetic model (48). Profiles of $M_{n}$ predicted by the model are shown in Fig. 9(d), further illustrating this.

**Table 1:** Thermal and chemical parameters for PLA.

Parameter	Unit	Value	Physical significance
$D_{w}$	m${}^{2}$ day^−1^	$7.5\times 10^{-7}$	Diffusion coefficient of water
K	m${}^{3}$ mol^−1^ day^−1^	$7.4\times 10^{-8}$	Reaction rate constant
$C_{b,0}$	mol/m${}^{3}$	$1.72\times 10^{4}$	Initial concentration of ester bonds
$\chi_{0}$	–	4.6	Initial interaction parameter
α	–	10.3	Fitting parameter related to affinity
β	–	0.65	Fitting parameter related to affinity
$T_{g,\infty }$	K	330.2	Theoretical maximum $T_{g}$
B	K kg mol^−1^	120	Fitting parameter in the Flory-Fox equation
$\Delta T_{g,0}$	K	12	Maximum drop in $T_{g}$ due to water uptake
$\omega_{l}$	–	0.016	Water concentration at the transition point
η	–	0.88	Fitting parameter in the effective temperature model

### 6.2 Mechanical response of dry undegraded PLA

We next consider the response of undegraded dry PLA cylinders in uniaxial compression tests at constant true strain rate of 0.005 s^−1^ at different testing temperatures between 37 °C and 51 °C. One-eighth of the cylindrical geometry was modelled and discretised using 384 C3D8 elements. As shown in Fig. 8c, symmetry boundary conditions were applied to the three symmetric planes. A uniaxial displacement corresponding to a constant true strain rate was applied to the top surface along the height direction. The average stress was calculated as the ratio of the total reaction force by the current cross-section area. The total force was obtained by summing the reaction forces on all the nodes on the bottom surface. We used the incompressibility assumption to calculate the current area of cross section. Since the strain field is homogeneous, the problem again reduced to a one-dimensional one. A separate one-dimensional solution also obtained to validate the three-dimensional finite element implementation. The material parameters were determined as follows. The parameters $E_{0}^{\left(1\right)}$, $T_{E}$, and $\beta_{E}$ related to the Young modulus were determined by fitting the Young modulus-temperature curve, with the modulus determined from the slope of the experimental true stress–strain curve. The Poisson ratio was set to 0.35 (Pan et al., 2024) and was assumed invariant with temperature. The parameters V, ${\overset{˙}{\gamma }}_{0}$, $\Delta G_{0}$, $T_{\Delta G}$, and $\beta_{\Delta G}$ were determined by fitting the experimental upper yield stress points using the analytical approximation of the yield stress based on Eq. (39) under uniaxial compression:

(56) $${\left|\sigma_{y}\right|}_{Y}=\frac{\sqrt{3}}{V}\left(\ln\left|\frac{{\overset{˙}{\epsilon }}_{y}}{{\overset{˙}{\gamma }}_{0}}\right|+\ln\sqrt{6}\right)k_{B}T+\frac{\sqrt{3}}{V}\Delta G$$

where $\epsilon_{y}$ and $\sigma_{y}$ denote the true axial strain and stress along the loading (y) direction. The remaining parameters related to softening ($H_{0}^{s}$, $T_{H}$, and $\beta_{H}$) and rehardening ($E_{0}^{\left(2\right)}$ and $J_{m}$) were obtained by fitting the post-yield portion of the stress strain curves. All the parameters are collected in Table 2. Therefore, while the total number of parameters is large, each group of parameters is constrained by distinct physical observations. The total number of parameters is also consistent with state-of-the-art constitutive models for glassy polymers, e.g. Dupaix and Boyce, 2007, Ames et al., 2009, Srivastava et al., 2010, Poulain et al., 2014, Wang et al., 2021 and Zhao et al. (2025). The same parameters were used in the simulation of wet degraded samples in the next section without further fitting.

**Fig. 10:**
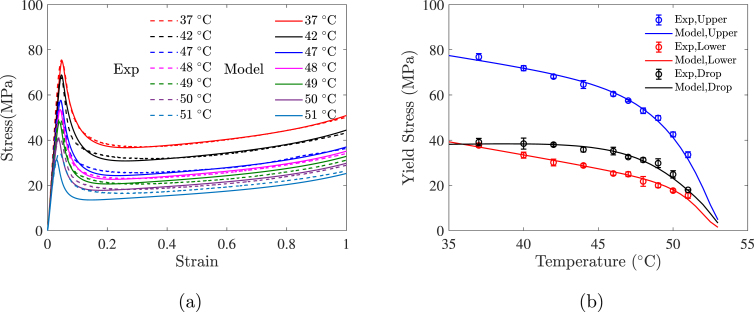
Comparison between model predictions and experimental results for (a) stress strain curves and (b) yield stress for dry undegraded PLA at different temperatures.

Stress–strain curves for the undegraded PLA at different temperatures are shown in Fig. 10(a). The model well reproduces the mechanical response across all temperatures. Notably, it effectively captures the temperature-dependent yield stresses and temperature-dependent softening behaviour of PLA (Fig. 10(b)). It should however be noted that the good agreement between the model and the experiments does not constitute a complete validation, since all experimental data were used for calibration. Complete validation would require additional experimental data covering a broader range of loading conditions in terms of prescribed thermo-mechanical loading history, including unloading.

### 6.3 Mechanical response of wet degraded PLA

**Fig. 11:**
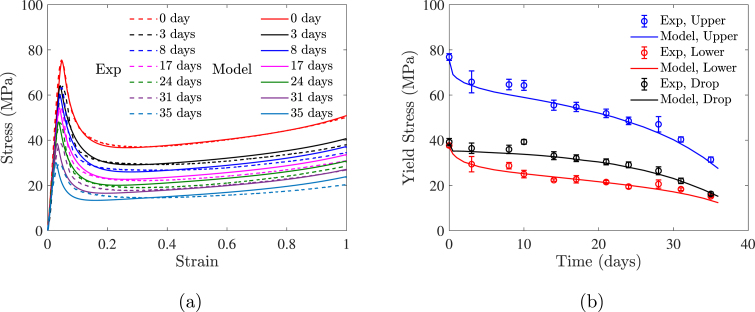
Comparison between model predictions and experimental results for (a) stress strain curves and (b) yield stress for wet degraded PLA after different periods of degradation.

Finally, we consider the deformation of wet degraded PLAs after different degradation times. Model predictions were obtained by simulating a compression test on one-eighth of the cylindrical geometry, accounting for pre-existing heterogeneous profile of water concentration and molecular weight, obtained in prior degradation simulation as explained in Section 6.1. The simplified geometry was discretised into 384 C3D8 elements. The boundary conditions were the same as in Section 6.2. At this point, the only remaining parameter to be identified is the parameter η in the effective temperature model (43), which was determined by fitting of the stress–strain curves after degradation on the experimental data. The best-fit value of η was identified as η=0.88, which is close to one, indicating that the effect of degradation on mechanical properties can be well captured by raising the testing temperature of the dry undegraded polymer by an amount close to the glass transition drop in the wet degraded polymer. The comparison between numerical predictions and experimental results is shown in Fig. 11. Overall, the effective temperature model provides a reasonable prediction of the overall stress–strain response, including yielding and softening. A notable deviation can however be seen at large deformations, with degradation causing a more pronounced loss of hardening capacity than predicted by the effective temperature model. This could be due to the fact that the strain hardening response is not solely governed by the kinetics of polymer segments, but also by entropic and energetic effects associated with chain stretching, which are not expected to follow a temperature-degradation equivalence.

**Table 2:** Mechanical parameters for PLA.

Parameter	Unit	Value	Physical significance
$E_{0}^{\left(1\right)}$	MPa	1900	Young’s modulus in the viscoplastic branch
$\nu^{\left(1\right)}$	–	0.35	Poisson’s ratio in the viscoplastic branch
$T_{E}$	K	331.2	Reference temperature for the modulus
$\beta_{E}$	–	50	Fitting parameter for the modulus
$E_{0}^{\left(2\right)}$	MPa	12	Young’s modulus in the hardening branch
$\nu^{\left(2\right)}$	–	0.45	Poisson’s ratio in the hardening branch
$J_{m}$	–	6	Parameter describing the maximum extensibility of the hardening branch
$\Delta G_{0}$	J	$7.33\times 10^{-19}$	Activation energy at absolute zero
$T_{\Delta G}$	K	331.6	Reference temperature for the activation energy
$\beta_{\Delta G}$	–	115	Fitting parameter for the activation energy
$H_{0}^{s}$	–	20	Steady-state yield strength-related parameter
$T_{H}$	K	325	Reference temperature for steady-state yield strength-related parameter
$\beta_{H}$	–	150	Fitting parameter for steady-state yield strength-related parameter
h	–	250	Softening parameter
${\overset{˙}{\gamma }}_{0}$	s^−1^	$3.50\times 10^{56}$	Reference flow rate
V	mm${}^{3}$	$3.64\times 10^{-18}$	Activation volume

## 7 Numerical examples

### 7.1 Diffusion-limited vs. reaction-limited degradation

In this section, we illustrate the model capabilities in capturing the transition between reaction-limited degradation and diffusion-limited degradation, depending on the specimen size, reaction rate, and diffusion coefficient. To this end, we consider again the degradation of a long rod, so that the only relevant geometry parameter is the rod radius R. We first introduce a dimensionless parameter $\overset{¯}{\tau }=\frac{\tau_{d}}{\tau_{r}}=\frac{R^{2}KC_{b,0}}{D_{w}}$, in which $\tau_{d}=\frac{R^{2}}{D_{w}}$ denotes the characteristic time for diffusion and $\tau_{r}=\frac{1}{KC_{b,0}}$ denotes the characteristic time for reaction. The value of $\overset{¯}{\tau }$ in Section 6 is calculated as $2.72\times 10^{-2}$. In the examples below, the parameter $\overset{¯}{\tau }$ was changed by changing the radius R, keeping all the other parameters constant and given by their values identified in Section 6.

**Fig. 12:**
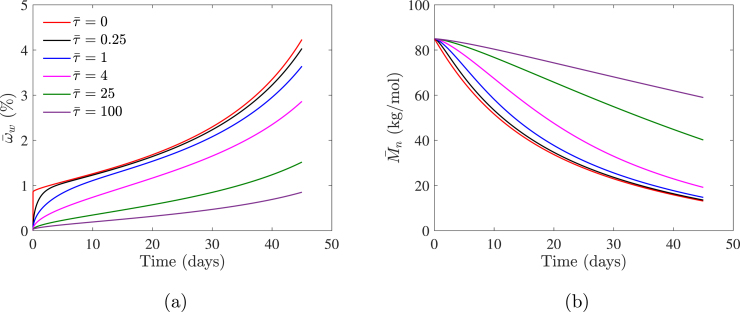
Effect of $\overset{¯}{\tau }$ on the time evolution of (a) average water uptake and (b) average number average molecular weight during degradation of a long rod.

Fig. 12(a) shows the evolution of the average water uptake as a function of time in long rods with $\overset{¯}{\tau }$ of 0, $6.80\times 10^{-3}$, $2.72\times 10^{-2}$, $1.09\times 10^{-1}$, $6.80\times 10^{-1}$, and 2.72, corresponding to rod radii of 5,10,20,50, and, 100mm, respectively. The case $\overset{¯}{\tau }=0$ corresponds to infinitely fast diffusion, i.e. homogeneous degradation. The smaller $\overset{¯}{\tau }$, the faster the water uptake by diffusion, with the water uptake instantaneously reaching the equilibrium water uptake in the undegraded polymer in the case $\overset{¯}{\tau }=0$. Subsequent increase in water uptake is due to degradation-induced increase in affinity, as previously discussed. Fig. 12(b) shows the corresponding evolution of the volume-average number average molecular weight, with faster water uptake (lower $\overset{¯}{\tau }$) leading to faster degradation. Notably, the influence of $\overset{¯}{\tau }$ becomes negligible when $\overset{¯}{\tau }\leq 6.80\times 10^{-3}$. Above this (approximate) value, the degradation mechanism gradually shifts from bulk degradation with nearly homogeneous concentration profile to surface degradation.

**Fig. 13:**
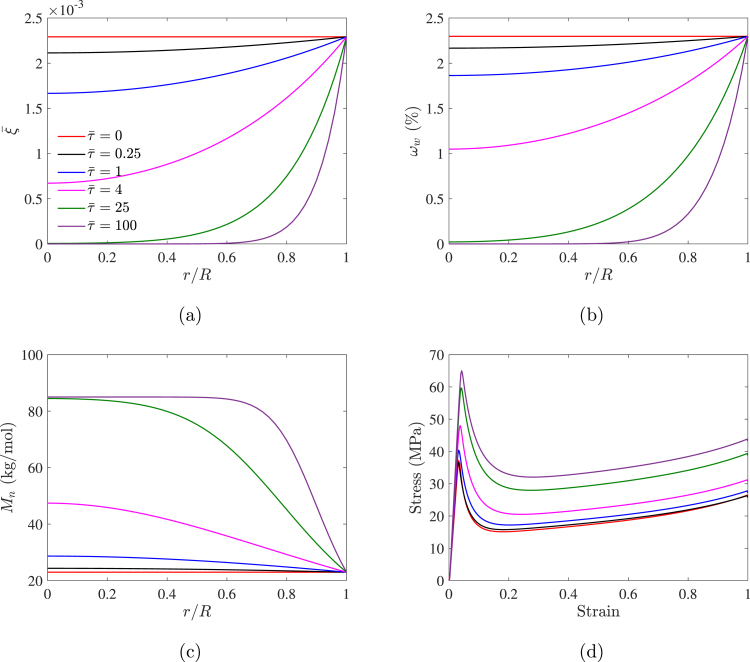
Profiles of (a) extent of reaction, (b) water content and (c) number average molecular weight along the radius for different $\overset{¯}{\tau }$ after 30 days of degradation. (d) True compression stress–strain curves after 30 days degradation for different $\overset{¯}{\tau }$.

The radial profiles of normalised extent of reaction, water uptake and number average molecular weight after 30 days of degradation are shown in Figs. 13(a), 13(b), and 13(c). For small values of $\overset{¯}{\tau }$, the extent of reaction and water uptake remain nearly uniform across the radius, indicating bulk degradation controlled by rapid diffusion. As $\overset{¯}{\tau }$ increases, pronounced gradients develop, with both water concentration and reaction extent becoming increasingly localised near the surface, while the core remains weakly degraded. Correspondingly, the number-average molecular weight exhibits strong radial heterogeneity for large $\overset{¯}{\tau }$, with severe molecular weight reduction near the surface and a relatively intact interior. It should be noted that heterogeneous water uptake does not induce any stress in the model in the absence of externally-applied loads, since we have neglected any swelling due to water uptake. These heterogeneous degradation profiles directly influence the mechanical response of the pre-degraded polymer shown in Fig. 13(d). Increasing $\overset{¯}{\tau }$ leads to higher apparent stiffness due to the preserved core, whereas smaller $\overset{¯}{\tau }$ results in more uniformly degraded material and reduced mechanical performance.

Predicted profiles of water uptake and extent of degradation presented in this section primarily illustrate the interplay between reaction-limited and diffusion-limited processes. Predicted profiles for the extent of degradation could also be used to predict the profile of elastic modulus and strength. In principle, profiles of mechanical properties could then be compared to direct experimental measurements obtained by nanoindentation or AFM (Gu et al., 2001, Naseem et al., 2019, Melelli et al., 2021). However, AFM-based studies specifically addressing heterogeneous hydrolytic degradation through the polymer thickness remain limited, see Tian et al. (2023) and Mozgova et al. (2025) for recent reviews.

### 7.2 Degradation and deformation of porous structures

In this section, we use the model to simulate the degradation of porous structures, inspired by biomedical applications such as 3D-printed bone scaffolds (Grémare et al., 2018, Gayer et al., 2019, Bakhtiari et al., 2024). The overall geometry is a rectangular cuboid with a square array of through-thickness holes. The side length is L=50mm, while the height is set to H=10L, which is sufficiently large to eliminate end-surface effects and allows us to focus on the influence of pore architecture on degradation and deformation. To this end, we varied the number of pores while keeping the porosity (defined as the volume ratio of the holes to the cuboid) constant (19.63%), resulting in radii of R=12.5mm, 6.25mm, 3.125mm, and 1.5625mm for geometries with 1, 4, 9, and 16 holes, respectively. For degradation simulations, the water concentration was prescribed on the outer surface of the cuboid and the hole surfaces, and degradation occured under no applied mechanical loads. Taking advantage of symmetry, only one-eighth of the 3-dimensional geometry was simulated, using symmetry boundary conditions on the three internal planes. The geometries were discretised using over 1600 C3D8 elements for all cases.

**Fig. 14:**
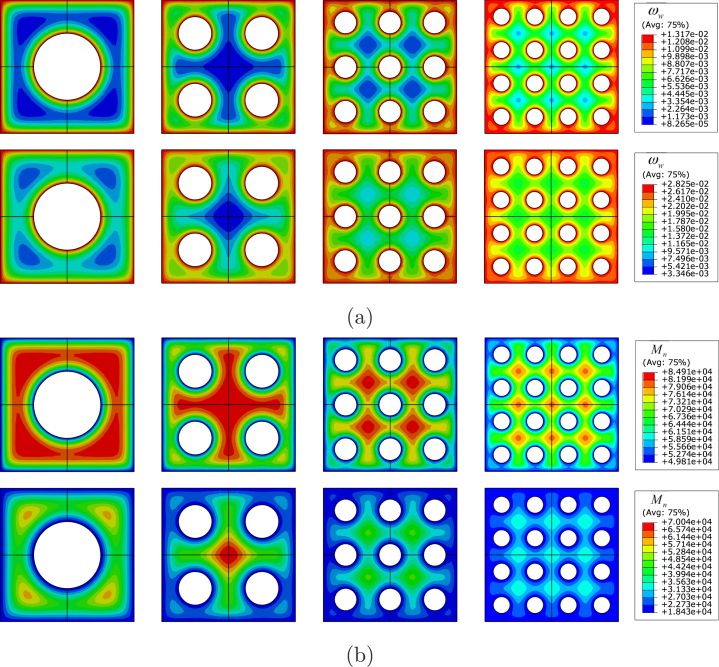
Effect of pore architecture on degradation behaviour of porous structures. Contours of (a) water fraction and (b) average molecular weight after 10 days (first row) and 35 days (second row) degradation for different designs.

The distributions of water fraction and molecular weight after 10 and 35 days are shown in Fig. 14. The pore geometry plays a dominant role in governing how degradation progresses within the porous structure interior. The water fraction distributions (Fig. 14(a)) reveal that interconnected pores enhance penetration pathways, leading to greater internal hydration and accelerated degradation, while more isolated pore arrangements confine water diffusion to local regions, producing spatially heterogeneous profiles. The molecular weight maps (Fig. 14(b)) further highlight the impact of pore architecture, with multi-pore structures promoting faster and more homogeneous molecular weight reduction, in contrast to the slower, surface-dominated degradation observed in single-pore structure.

**Fig. 15:**
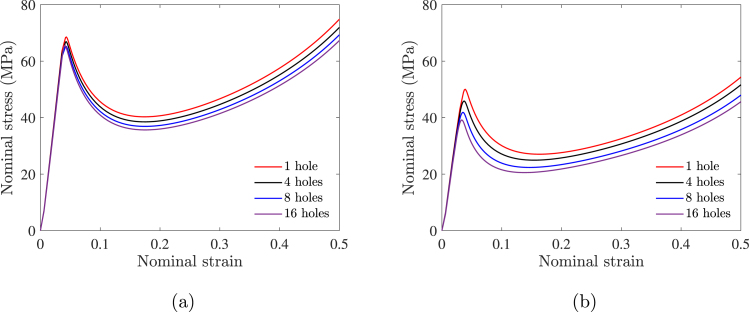
Effect of pore architecture on the compression response after (a) 10 days and (b) 35 days of degradation.

Next, we simulated the mechanical response of degraded porous structures by applying displacements corresponding to a constant nominal strain rate of 0.006 s^−1^ on the top surface while the normal displacements of the bottom and two adjacent internal surfaces was set to zero. External surfaces were traction free. Here, the nominal stress is defined as the total force divided by the cross-sectional area of the square, $L^{2}$, while the nominal strain is calculated as the displacement divided by the original height of the geometry. Fields of $C_{w}$ and ξ calculated after a pre-defined degradation period were used to determine the profile of glass transition temperature. The nominal compressive stress–strain curves after 10 and 35 days of degradation are shown in Fig. 15. The yield stress is lower for structures with more holes because of a larger average extent of degradation, and this difference becomes more pronounced after a longer degradation period. This can be attributed to larger overall extent of degradation for a structure with more holes. Overall, these results demonstrate that the spatial arrangement of pores impacts degradation kinetics by modulating water accessibility and reaction uniformity, which in turn influences the deformation behaviour, thereby highlighting pore architecture as a key design parameter for tailoring the properties of biodegradable structures.

### 7.3 Concurrent creep and degradation

During service, biodegradable devices are often subjected to constant or cyclic mechanical loads, under which time-dependent deformation (creep) may occur. In fact, previous experimental studies have demonstrated that PLA is prone to premature creep failure, well before significant hydrolytic degradation can take place (Smit et al., 2010, Engels et al., 2010, Dreher et al., 2014, Chen et al., 2026), and in particular before any ductile-to-brittle transition caused by chain scission. Creep occurs in the dry polymer, and is further accelerated in the presence of water and hydrolysis (Chen et al., 2026). Therefore, understanding the effect of water uptake and hydrolytic degradation on the creep response is essential to ensure that biodegradable devices perform reliably throughout their functional lifetime.

We consider a cylinder (height: 8 mm, diameter: 8 mm) immersed in water and subjected to a constant nominal stress (or force) on its top surface. We assume that water diffuses only through the cylinder lateral surface (i.e. the top and bottom of the cylinder are assumed impermeable). Accounting for symmetry, only one eighth of the geometry was discretised using 384 C3D8T elements. Symmetry boundary conditions were applied to the three adjacent symmetry planes. A rigid body was placed in contact with the top surface, with hard contact enforced in the normal direction and frictionless conditions in the tangential direction. A constant compressive force along the height direction was then applied to the rigid body.

**Fig. 16:**
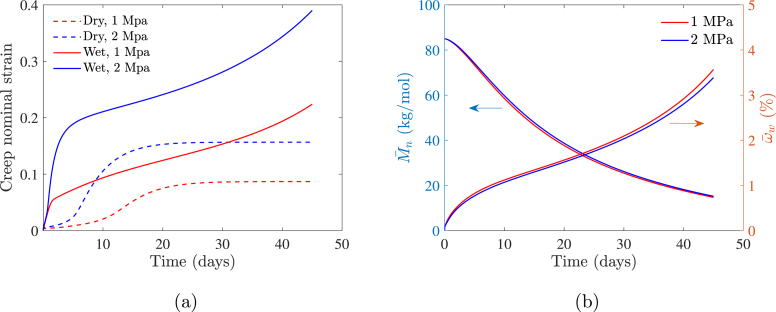
(a) Evolution of the compressive nominal creep strain in dry and immersed PLA cylinders subjected to a constant nominal stress at 45 °C. (b) Corresponding evolution of the volume-average water uptake and number average molecular weight.

Fig. 16(a) shows the time evolution of the compressive nominal creep strain in dry and immersed PLA cylinders subjected to nominal compressive stresses of 1MPa and 2MPa. Note that these stress values are significantly lower than the initial yield stress (approximately 61MPa). In the dry, undegraded cases, the creep deformation curve exhibits a reverse S-shape, which is similar to the findings reported by Dreher et al. (2014) for PLA and PLGA under a constant tensile stress. The sample initially responds with a small elastic deformation under low applied stress. This is followed by creep deformation at nearly constant strain rate until a few percent strain is reached. After that, the strain rate increases, then gradually decreases until a certain deformation level is reached, depending on the applied stress. The rapid increase in creep strain is attributed to material softening, i.e., mechanical rejuvenation. Finally, the deformation saturates, and the strain approaches a plateau with little to no further increase over time. We attribute this effect to the reduction in true stress due to enlarged bearing area and rehardening at large deformations.

In contrast, wet samples exhibit a markedly different deformation response. Following the initial elastic response, the strain increases rapidly with time, indicating accelerated creep and earlier softening compared to the dry condition. This is primarily attributed to water uptake, as evidenced by the rapid water uptake in the first few days (Fig. 16(b)) and the sharp decrease in $T_{g}$ with water for ${\overset{¯}{\omega }}_{w}<1\text{\%}$ (Fig. 7(b)). The contribution of degradation is negligible at this stage, since $T_{g}$ changes only weakly with molecular weight in the early degradation stage (Fig. 6(b)). For example, the volume-averaged molecular weight ${\overset{¯}{M}}_{n}$ decreases from 85 kg/mol to approximately 75 kg/mol during the first five days, which causes a drop in $T_{g}$ of less than 0.2°C. After a few days, the strain rate begins to decrease, likely due to the increased cross-section area. Over a prolonged period, the deformation continues to accumulate without reaching a clear plateau, which can be attributed to the progressive reduction in molecular weight further enhancing chain mobility and sustaining long-term creep.

Fig. 16(b) shows the time evolution of water absorption and molecular weight under different stress levels. Higher stress induces greater deformation, which increases the diffusion path length for water and consequently reduces the degradation rate. However, the observed differences are minimal because the small specimen radius results in a short diffusion time for water.

Overall, these findings imply that prolonged in vivo loading could lead to permanent deformation and loss of shape stability, even in applications where applied loads are well below the material’s nominal strength.

## 8 Conclusions

We have developed a constitutive model for concurrent hydrolytic degradation and viscoplastic deformation in glassy polymers degrading by hydrolysis. An effective temperature approach was used to describe the effect of water uptake and reduction in molecular weight on the mechanical properties, enabling the prediction of the deformation behaviour of wet, degraded polymers based on a description of the response of the dry undegraded polymer at elevated temperature. We have also conducted systematic experiments for amorphous PLA to calibrate our model. Comparison between the model predictions and experimental data showed that our model reasonably well reproduces all the experimental observations including the evolution of molecular weight, water absorption and deformation behaviour after degradation. We further illustrated the model capability in several case studies, exploring the effects of specimen size, porous structure, and concurrent creep and degradation.

Our modelling approach could be generalised in several directions. First, the effective temperature framework could in principle be used with other thermo-mechanical constitutive models for glassy polymers, which is a key advantage of the method. In particular, it would be desirable to use constitutive models with fewer fitting parameters and phenomenological functions. The development of such theories remains an active area of research, even in the absence of degradation (Chevalier et al., 2018). Next, we have assumed that inelastic deformations are governed by (volume-preserving) shear yielding, and did not account for dilatational plasticity due to possible crazing or porosity development under tension. While the effective temperature framework can in principle accommodate constitutive models accounting for dilatational plastic flow, further experimental studies are needed to characterise the elasto-viscoplastic response of degrading polymers under various triaxiality ratios. Third, the theory could be generalised to describe two-way couplings, including a description of swelling induced by water uptake and possible effect of stress on water transport and kinetics of hydrolysis, building on recent theoretical contributions (Loeffel and Anand, 2011, Konica and Sain, 2020, Pan and Brassart, 2022, Alkhoury and Chester, 2025). Additional experimental data or atomistic simulations are needed to inform the formulation of specific constitutive models describing coupled effects. Further generalisations could include a description of mass loss by the release of short chains, degradation-induced recrystallisation, and autocatalysis due to the trapping of acidic reaction products. Finally, the constitutive model for deformation and degradation should be supplemented by a description of the ductile-to-brittle transition. Such generalisations would extend the range of application of the model to a broader degradation window and a broader class of glassy biodegradable polymers.

## CRediT authorship contribution statement

**Zhouzhou Pan:** Methodology, Investigation, Formal analysis, Software, Writing – original draft. **Huanming Chen:** Methodology, Investigation, Formal analysis, Writing – original draft. **Laurence Brassart:** Conceptualization, Methodology, Formal analysis, Supervision, Funding acquisition, Writing – original draft, Writing – review & editing.

## Declaration of competing interest

The authors declare that they have no conflict of interest.

## Data Availability

The Abaqus subroutines implementing the model are available on Github (Pan and Brassart, 2026).
